# Effects of Double-Ageing Heat Treatments on the Microstructure and Mechanical Behaviour of a Ti-3.5Al-5Mo-4V Alloy

**DOI:** 10.3390/ma14010209

**Published:** 2021-01-04

**Authors:** Xuanming Ji, Panpan Ge, Song Xiang, Yuanbiao Tan

**Affiliations:** 1 College of Materials and Metallurgy, Guizhou University, Guiyang 550025, China; xmji@gzu.edu.cn (X.J.); panpange1995@126.com (P.G.); ybtan1@gzu.edu.cn (Y.T.); 2 Key Laboratory for Mechanical Behavior and Microstructure of Materials of Guizhou Province, Guizhou University, Guiyang 550025, China

**Keywords:** Ti-3.5Al-5Mo-4V alloy, double ageing, phase transition, hardness

## Abstract

In this work, the effect of double-ageing heat treatments on the microstructural evolution and mechanical behaviour of a metastable β-titanium Ti-3.5Al-5Mo-4V alloy is investigated by X-ray diffraction (XRD), scanning electron microscopy (SEM) and transmission electron microscopy (TEM). The double-ageing treatments are composed of low-temperature pre-ageing and high-temperature ageing, where the low-temperature pre-ageing is conducted at 300 °C or 350 °C for different times, and the high-temperature ageing is conducted at 500 °C for 8 h. The results show that the phase transformation sequence is altered with the time spent during the first ageing stage, the isothermal ω phase is precipitated in the pre-ageing process of the alloy at 300 °C and 350 °C with the change in the ageing time, and the ω phase is finally transformed into the α phase with the extension of pre-ageing time. The existence time of the ω phase is shortened as the pre-ageing temperature increases. The microhardness of the alloy increases with increasing pre-ageing time and temperature. Compared with single-stage ageing, the ω phase formed in the pre-ageing stage changes the response to subsequent high-temperature ageing. After the two-stage ageing treatment, the precipitation size of the α phase is obviously refined after the double-ageing treatment. A microhardness test shows that the microhardness of the two-stage aged alloy increases with extended pre-ageing time.

## 1. Introduction

Metastable β-titanium alloys are widely used in aerospace fields such as aircraft structures, rockets and satellites because of their prominent properties, which include high specific strength, low elastic modulus and corrosion resistance. The development of new metastable titanium alloys has become a key focus in the development of new generations of weapons and equipment [1,2,3,4,5,6,7,8]. In recent years, the Northwest Institute of Nonferrous Metals has designed a new type of Ti-Mo-V-Cr-Fe-Sn-Zr-Al high-strength metastable β-titanium alloy based on the principle of multiple strengthening under critical molybdenum-equivalent conditions; its nominal composition is Ti-3.5Al-5Mo-4V-2Cr-1Fe-2Zr-2Sn (Ti-3.5Al-5Mo-4V for short). Compared with Ti-38-6-44 (Ti-3Al-8V-6Cr-4Mo-4Zr), Ti-15-3 (Ti-15V-3Cr-3Sn-3Al) and other alloys, the Ti-3.5Al-5Mo-4V alloy has the highest ageing enhancement under the same ageing temperature. It is a new metastable titanium alloy with broad application prospects and good matching comprehensive properties (ultimate tensile strength: 1200–1500 MPa; elongation: 8%–18%) [9,10].

The main strengthening of the near β-titanium alloy and metastable β-titanium alloy derives from the secondary α phase being precipitated from the metastable β phase during the ageing treatment in the (α + β) two-phase region. The morphology, distribution, size and volume fraction of the secondary α phase precipitated in the β matrix determine the mechanical properties and serviceability of the titanium alloy, and these elements can be effectively adjusted by changing the heat treatment temperature, time, heating/cooling rate and other heat treatment factors [11,12,13].

Ageing heat treatment is a common means to enhance the performance of titanium alloys; however, previous studies have shown that a single-stage ageing heat treatment can form a no-precipitation zone and grain boundaries with equal inhomogeneous structures in the alloy. This will cause uneven hardening of the material and inhomogeneous strain when loaded, which will eventually reduce the strength and plasticity of the alloy and affect its potential application. In recent years, a new type of double-stage ageing heat treatment different from single-stage heat treatment has attracted attention. Compared with the traditional single-stage ageing process, double-stage ageing can significantly refine the precipitation phase, avoid a no-precipitation zone and improve the mechanical properties of type β-titanium alloy [14,15,16]. Ivasishin et al. made the precipitation of the α phase more homogeneous through simple double-stage ageing and improved the mechanical properties of Ti15-3 titanium alloy; compared with single ageing, the tensile strength and elongation after fracture of the alloy were improved at the same time [7,17]. When Schmidt studied the phase transformation of β-C titanium alloy during ageing, it was found that the isothermal ω phase could be precipitated in the alloy at low-temperature pre-ageing at 350 °C. The isothermal ω phase provided a nucleation point for the α phase, which provided a good basis for the precipitation uniformity and size refinement of the α phase; on the other hand, the isothermal ω phase can also inhibit the transformation of the β phase to the α” phase, thereby reducing the loss of strength and hardness [18]. Santhosh R.’s research on a Ti-15V-3Cr-3Al-3Sn alloy showed that fine precipitates can be obtained by pre-ageing at 250 °C and 350 °C before high-temperature ageing, ultimately obtaining a fine precipitated phase with a homogeneous distribution [19].

The existing research shows that the secondary phases can be effectively refined by double ageing; however, research on the influences of double ageing on the microstructure and mechanical properties of titanium alloys has mainly paid attention to the pre-ageing temperature, and there are few studies on the effects of pre-ageing time, especially double-stage ageing research on metastable β-titanium alloys, which is even rarer [17,20,21,22]. As a new type of metastable β-titanium alloy, the phase transformation mechanism of the Ti-3.5Al-5Mo-4V alloy is similar to that of the same type of metastable β-titanium alloy; however, the particularity of its critical value molybdenum equivalent inevitably leads to its phase transformation mechanism being different from that of a general metastable β-titanium alloy. At the same time, the current research on Ti-3.5Al-5Mo-4V alloys has mainly focused on thermal deformation and single-stage ageing heat treatment [23,24]. To improve the mechanical properties of Ti-3.5Al-5Mo-4V alloys and expand their application in aerospace and other fields, it is necessary and significant to study the rule of microstructure evolution during the double-stage aging process. Therefore, in this study, a Ti-3.5Al-5Mo-4V alloy was taken as the research object, and a double-stage ageing heat treatment was carried out to explore the method of obtaining a finely dispersed and homogeneously distributed precipitation phase in this titanium alloy. Furthermore, the evolution of the microstructure and mechanical properties of the metastable β-titanium alloy during the double-stage ageing process was revealed.

## 2. Experimental Materials and Methods

The material studied in this experiment is a Ti-3.5Al-5Mo-4V alloy prepared by vacuum arc remelting technology, and its chemical composition is displayed in Table 1. The transformation temperature of the alloy is 820 ± 5 °C, as determined by the metallographic method.

**Figure 1:**
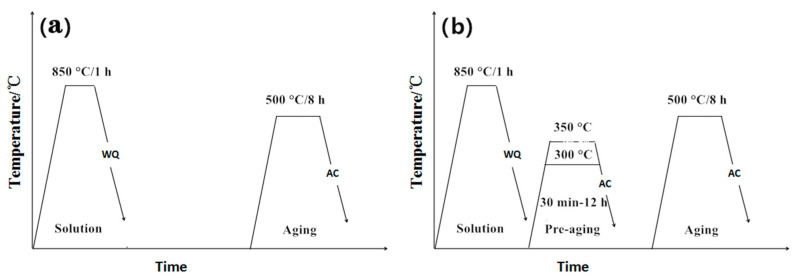
The schematic illustration of heat treatment routine for the Ti-3.5Al-5Mo-4V alloy. (**a**) Single-stage ageing. (**b**) Double-stage ageing.

The alloy was subjected to solution treatment at 850 °C/1 h to eliminate forging stress and obtain a uniform single β phase structure, and then it was water-quenched (WQ) to room temperature. To study the microstructural evolution of the alloy during double ageing, the samples after solution treatment were isothermally aged at 300 °C and 350 °C in a salt bath furnace for 5 min to 12 h and then air-cooled (AC) to room temperature. Then, the low-temperature ageing samples were subjected to high-temperature ageing treatment at 500 °C in a salt bath furnace for 8 h and then air-cooled to room temperature. To compare with the double-ageing process, some samples were selected for single-stage ageing heat treatment at 500 °C/8 h. The schematic illustration of heat treatment is shown in Figure 1.

To analyse the microstructural evolution of the Ti-3.5Al-5Mo-4V alloy during the two-stage ageing process, the phase change during the ageing process was characterised by X-ray diffraction (XRD), and the pre-structure morphology was observed by scanning electron microscopy (SEM) and transmission electron microscopy (TEM).

The metallographic specimens were mechanically polished using a standard metallographic procedure and etched using Kroll’s reagent (1:2:7, HF:HNO_3_:H_2_O). The micrographs of the metallographic samples were generated by an OLYMPUS (Tokyo, Japan) GX51 optical microscope (OM). The ZEISS (Oberkochen, Germany) SUPRA40 field emission scanning electron microscope (FESEM) was used on the ageing-treated specimens to obtain the microstructures at 10 kV under InLens mode with a protective pure nitrogen atmosphere at room temperature.

The phase identification analysis of the Ti-3.5Al-5Mo-4V alloy was carried out using X-ray diffraction (XRD). XRD was conducted by the use of the Bruker D8 Advance (Karlsruhe, Germany) by CuKα radiation (λ = 1.5406 Å) at 40 kV and 40 mA at room temperature.

After heat treatment, thin slices were cut and ground to 50–60 mm. Then, the thin slices were cut into 3 mm small discs by a punching device and finally electropolished at 20 VDC at a temperature of 10 °C. A Tecnai G2 F20 transmission electron microscope from FEI (Hillsboro, OR, USA) was used for analysis and testing in the experiment, and the accelerating voltage was selected as 200 kV.

The Vickers micro-hardness was tested using an HVS-1000 digital microhardness measurement machine with a 0.98 N loading weight and 15 s loading time. All samples were wet-ground to 2000 grit and polished for testing. To ensure the reliability of the experiment, there were at least seven points measured for each specimen, and the average value was taken to reduce the experimental error.

## 3. Results and Discussion

### 3.1. Microstructure and Phase Composition of the Alloy after Solid Solution Treatment

**Figure 2:**
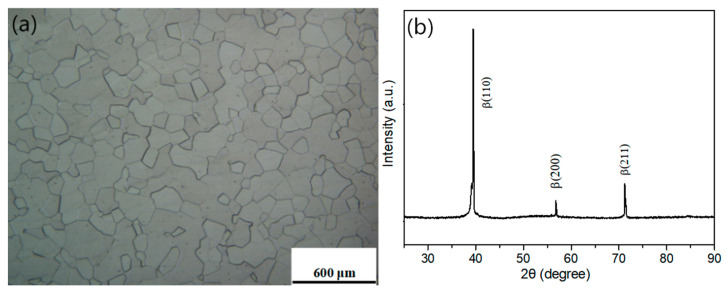
Microstructure and XRD pattern of the samples after solution treatment at 850 °C for 1 h. (**a**) OM. (**b**) XRD patterns.

Figure 2 shows the metallographic structure and XRD pattern of the Ti-3.5Al-5Mo-4V alloy after solid solution treatment at 850 °C/1 h. Figure 2a shows that the microstructure of the samples after solid solution treatment is mainly equiaxed β-phase grains with an average diameter grain of approximately 150–200 µm, indicating that the alloy is completely recrystallised after solid solution treatment. It can be seen in Figure 2b that there are only three β-phase diffraction peaks in the XRD pattern, indicating that the alloy is composed of the β-phase of the body-centred cubic structure after solid solution treatment; that is, after quenching, all β-phases are retained at room temperature.

### 3.2. Phase Precipitation and Tissue Evolution during the Low-Temperature Pre-Ageing Process

**Figure 3:**
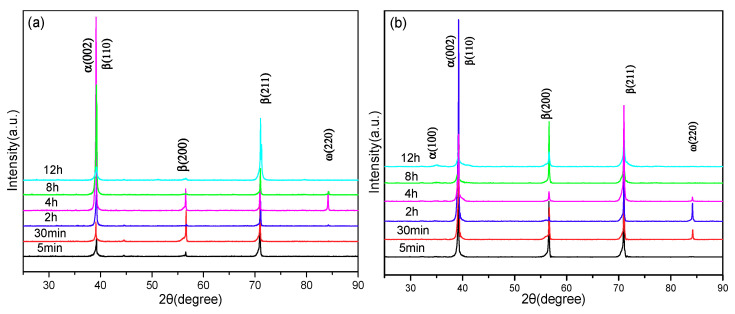
XRD patterns of alloy with different pre-ageing treatments after 850 °C/1 h solution: (**a**) 300 °C. (**b**) 350 °C.

Figure 3 indicates the XRD patterns of the alloy aged at 300 °C and 350 °C for 5 min, 30 min, 2 h, 4 h, 8 h and 12 h after solution treatment at 850 °C/1 h. As shown in Figure 3a, only the β phase existed when the pre-ageing time was 5 min at 300 °C, and a small quantity of ω phase was produced when the pre-ageing time was extended to 2 h. By continuing to extend the pre-ageing time to 4 h, the precipitation content of the isothermal ω phase continues to increase, but as the pre-ageing time is further extended, the precipitation content of the isothermal ω phase decreases, and when the pre-ageing time is extended to 12 h, the ω phase disappears. It can be shown in Figure 3b that after pre-ageing at 350 °C for 5 min, the phase composition of the alloy is similar to the result of pre-ageing at 300 °C with only the β phase. However, when the pre-ageing time is extended to 30 min, the ω phase precipitates in the alloy because the driving force of ω phase precipitation increases with increasing ageing temperature, which promotes the precipitation of the ω phase. With the prolongation of pre-ageing time, the precipitation content of the ω phase continues to increase and reaches a peak value at 2 h. As the pre-ageing time is extended, the precipitation content of the ω phase decreases. When the pre-ageing time increases to 8 h, the ω phase disappears, but the diffraction peak of the α phase can be observed in the spectrum at this time. This is due to the transition from the β phase to the ω phase during the low-temperature pre-ageing process, but the isothermal ω phase is an unstable intermediate phase, and its Gibbs free energy is significantly higher than that of the α phase. As the ageing time increases, the ω phase transforms into the α phase. When the pre-ageing time is extended to 12 h, the precipitation amount of the phase continues to increase, and the alloy is composed of α + β dual phases.

**Figure 4:**
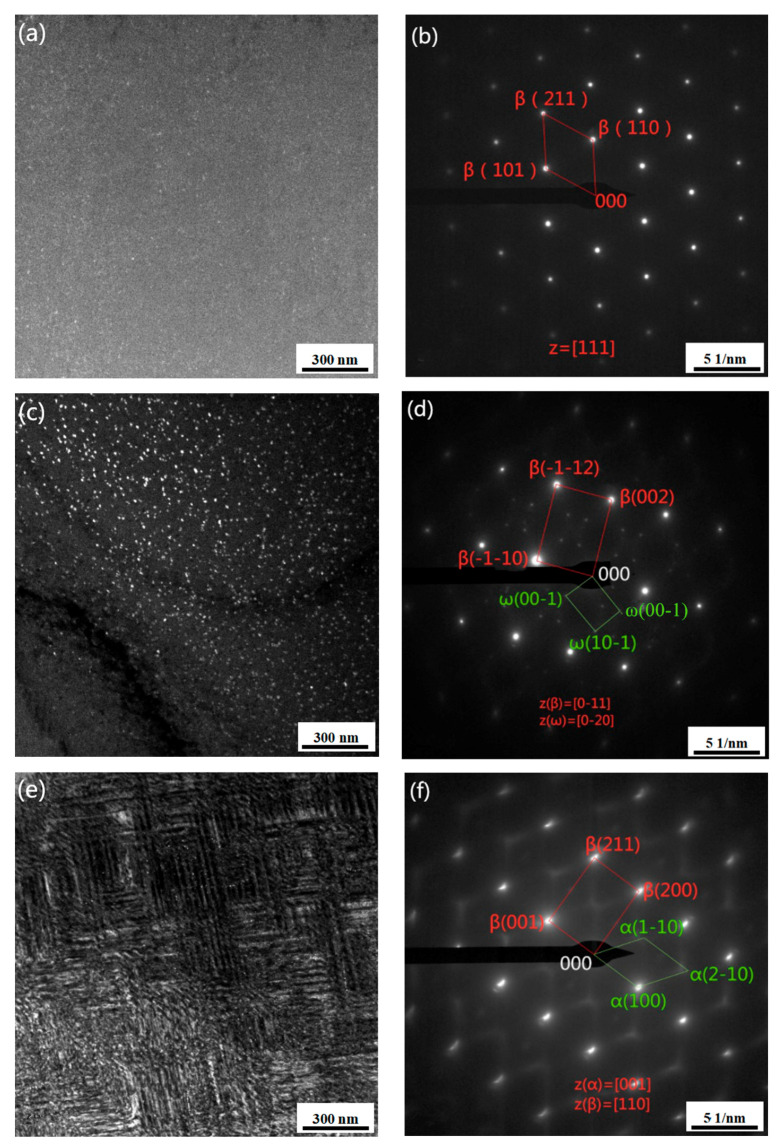
TEM of alloy pre-aged at 300 °C for different times: (**a**) TEM DF images of 2 h, (**b**) selected area electron diffraction (SAED) patterns of 2 h, (**c**) TEM DF images of 4 h, (**d**) SAED patterns of 4 h, (**e**) TEM DF images of 8 h and (**f**) SAED patterns of 8 h.

To further analyse the morphology and distribution of the isothermal ω phase, the microstructure of the Ti-3.5Al-5Mo-4V alloy pre-aged at low temperature was observed by transmission electron microscopy (TEM). Figure 4 displays the TEM morphology of the alloy after pre-ageing at 300 °C for different times. It can be shown in Figure 4a,b that fine precipitates are diffusely dispersed in the matrix after pre-ageing at 300 °C for 2 h; however, because the precipitates are too fine and their number is small, weak diffraction information is concealed by the matrix, so only the β matrix can be marked in the selected area electron diffraction (SAED) spots.

**Figure 5:**
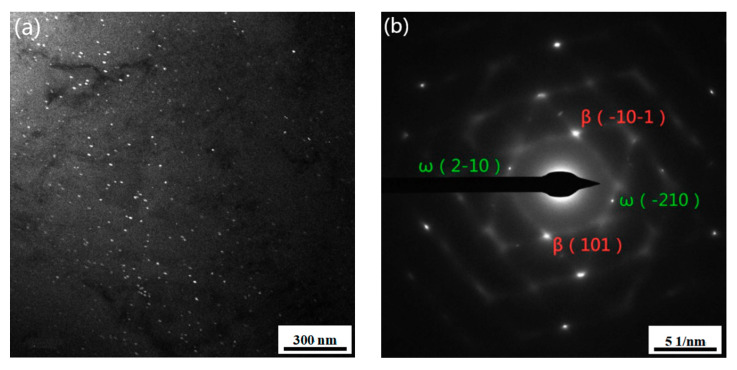
The TEM of alloy of pre-ageing at 300 °C for 2 h: (**a**) TEM DF images and (**b**) SAED patterns.

It can be found out in Figure 4c,d that a good deal of precipitates are diffusely dispersed in the matrix after 300 °C/4 h pre-ageing, and the size increases (approximately 15 nm) compared with 300 °C/2 h pre-ageing, while the analysis of SAED spots shows that the precipitated phase is a single ω phase, and its diffraction spots appear at 1/3{-1-12}β and 2/3{-1-12}β of the β matrix diffraction spots. It can be perceived in Figure 4e,f that the precipitated phase changes significantly when the pre-ageing time is prolonged to 8 h. At this time, the precipitates are mainly α phase-like needles with widths of approximately 50–100 nm. In other words, with the extension of ageing time, the transformation from ω phase to α phase occurs. Figure 5 indicates the TEM morphology of the alloy at 350 °C/2 h pre-ageing, and the comparison of the morphology with that at 300 °C/2 h pre-ageing shows that the amount and size of precipitates increase with pre ageing at 350 °C.

From the phase change and microstructure evolution, it can be considered that the β → α phase transition cannot be satisfied at low temperature pre-ageing due to the high potential barrier; therefore, the metastable intermediate ω phase is preferentially precipitated from the β matrix. With the extension of ageing time, the α-stable supersaturated element gradually spreads to the ω phase nucleation position, the ω phase gradually increases and the composition of the ω phase changes greatly at the same time. The ω → α phase transition occurs when the ageing time is extended to a certain extent. At the same time, the time node and existence time of isothermal ω phase transition are also different after different pre-ageing temperatures, the conditions of phase transition are easier to meet and the precipitation of the ω phase more easily occurs with the increase of the pre-ageing temperature.

### 3.3. Analysis of the Hardening Curve of the Alloy after Pre-Ageing Treatment

**Figure 6:**
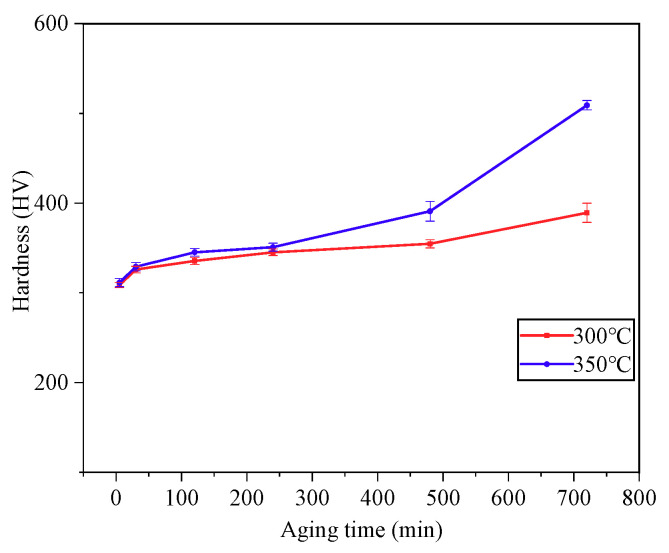
Microhardness of alloy with different pre-ageing treatments after 850 °C/1 h solution.

Figure 6 indicates the microhardness changes of the solid solution Ti-3.5Al-5Mo-4V alloy after various pre-ageing treatments. It can be shown in Figure 6 that with the increase in pre-ageing temperature, the alloy phase transformation kinetic conditions are sufficient, and the number of precipitated phases increases. Therefore, as the pre-ageing temperature increases, the hardness of the alloy increases; however, the solid-state transformation requires a certain reaction time. When the pre-ageing time is short, there is almost no precipitation of the ω phase, so the hardness barely changes. After pre-ageing at 300 °C, the hardness of the alloy increases with the increase in the precipitation phase as the ageing time increases; the variation trend of alloy hardness after pre-ageing at 350 °C is similar to that at 300 °C, the hardness of the alloy is increased, and the increasing trend is ever more obvious because the ω phase is constantly transformed and the α phase is precipitated; the hardness reaches 509 HV (HV0.1) at 12 h.

### 3.4. Microstructural Evolution of Double Ageing

**Figure 7:**
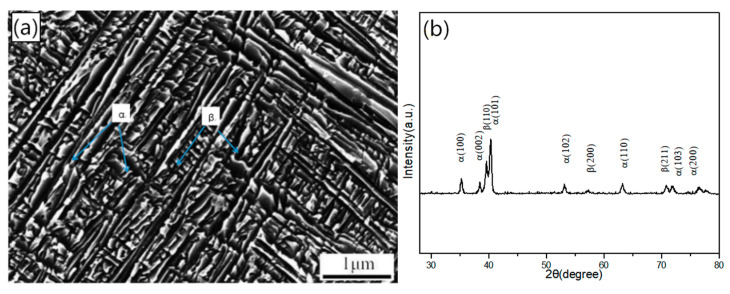
SEM and XRD patterns of the solid solution Ti-3.5Al-5Mo-4V alloy with ageing treatment at 500 °C/8 h. (**a**) OM. (**b**) XRD patterns.

The solid solution Ti-3.5Al-5Mo-4V alloy is subjected to a single-stage ageing treatment at 500 °C/8 h to compare and study the evolution of the ascending double-ageing structure. Figure 7 illustrates the microstructure and XRD pattern of the alloy after ageing at 500 °C/8 h. Figure 7 shows that the alloy is consists of the α + β phase at this time.

**Figure 8:**
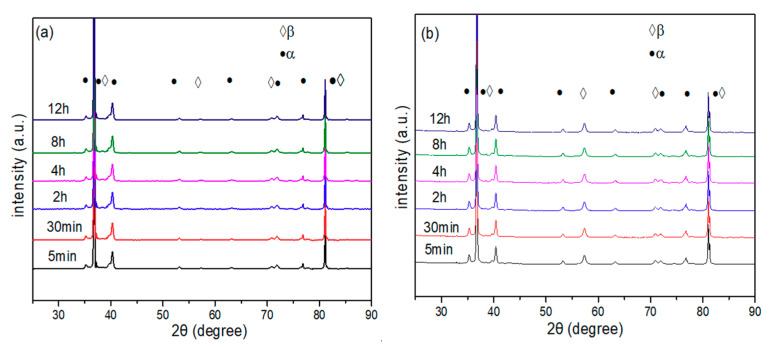
The XRD patterns of the solid solution Ti-3.5Al-5Mo-4V alloy after double-ageing treatment: (**a**) 300 °C and (**b**) 350 °C.

Figure 8 shows the XRD patterns of the solid solution Ti-3.5Al-5Mo-4V alloy, which is pre-aged at 300 °C and 350 °C for various times and then isothermally aged at 500 °C/8 h. As can be seen from the figure, the ω phase of the pre-aging precipitation disappeared, and a large number of secondary phases were precipitated out. Finally, the phase composition of the alloy after the double-stage aging heat treatment was α + β.

**Figure 9:**
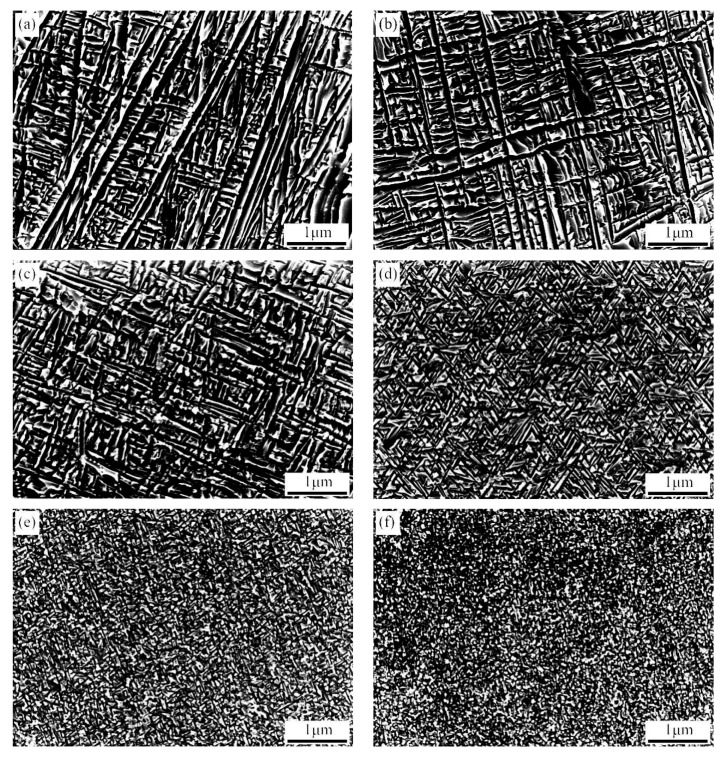
SEM of the solid solution alloy treated by double ageing and pre-aged at 300 °C for different times: (**a**) 5 min, (**b**) 30 min, (**c**) 2 h, (**d**) 4 h, (**e**) 8 h, and (**f**) 12 h.

Figure 9 shows the microstructure of the solid solution Ti-3.5Al-5Mo-4V alloy, which is pre-aged at 300 °C for various times and then isothermally aged at 500 °C/8 h. Compared with single-stage ageing, the needle-like secondary α phase in the microstructure after the 300 °C/5 min + 500 °C/8 h double-ageing treatment is refined to a certain extent, and a similar phenomenon is also found in the Ti-15Mo-2.7Nb-3Al-0.2Si alloy [25]. This is mainly due to the isothermal ω phase being produced in the low-temperature pre-ageing stage for the secondary phase to provide more nucleating particles; however, the dispersed small isothermal ω phase inhibits the α phase from growing [26,27]. As the pre-ageing time is extended to 30 min, the morphology of the secondary α phase does not change, but the size is obviously refined because with the extension of the pre-ageing time, the number of isothermal ω phases increases, which increases the number of nucleation particles in the secondary α phase [28]. When the pre-ageing time reaches 2 h, the morphology of the secondary α phase changes slightly, and the size continues to decrease. When the pre-ageing time is extended to 4 h, the secondary α phase changes from slender needles to short needles, and the arrangement is more compact. When the pre-ageing time reaches 8 h, the transformation from the ω-phase to the α-phase is basically completed during the pre-ageing process; the growth and spheroidisation of the α phase mainly occur during the subsequent high-temperature ageing process. At this time, the secondary phase changes from short spiculate to irregular ellipsoid after double-stage ageing. The shape change of the α phase can be attributed to the synergistic effects of the surface energy and distortion energy [29]. When the pre-ageing time reaches 12 h, the morphology and size of the secondary α phase are similar to those pre-aged for 8 h. This is because the nucleation particles in the matrix no longer increase after the pre-ageing time exceeds 300 °C.

**Figure 10:**
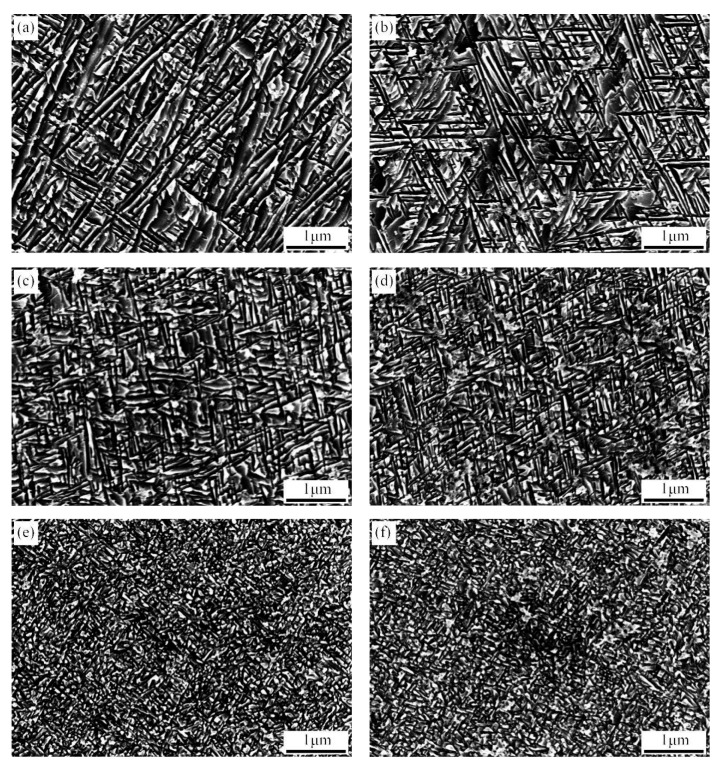
SEM of the solid solution alloy treated by double ageing and pre-aged at 350 °C for different times: (**a**) 5 min, (**b**) 30 min, (**c**) 2 h, (**d**) 4 h, (**e**) 8 h, and (**f**) 12 h.

Figure 10 shows the microstructure of the solid solution Ti-3.5Al-5Mo-4V alloy, which is pre-aged at 350 °C for various times and then isothermally aged at 500 °C/8 h. Its microstructural evolution law is similar to that of 300 °C pre-ageing. When the pre-ageing time is 5 min, the secondary α phase after ascending double ageing is short and slender, the structure is more homogeneous and the secondary α phase is smaller in size than in the 300 °C/5 min + 500 °C/8 h process. The secondary α phase size is further refined with the extension of the pre-ageing time to 30 min. When the pre-ageing time is extended to 4 h, the secondary α phase changes from slender to short, and the arrangement becomes more compact. After the pre-ageing time reaches 8 h, the short secondary α phase disappears and turns into an irregular shape, and as the pre-ageing time is extended, the morphology of the secondary α phase no longer changes, instead becoming a fine and uniform structure.

During the ascending double-ageing process of the alloy, in the pre-ageing stage, an α-stable element segregation zone is formed in the matrix, forming an isothermal ω phase and a fine secondary α phase. During the subsequent ageing process at 500 °C/8 h, the secondary α phase is precipitated out quickly in the segregation zone of the α stabilising elements, and a fine and uniformly dispersed secondary α phase is obtained [30]. The structure of the alloy can be controlled effectively by using the ascending double-ageing process.

### 3.5. Analysis of the Hardening Curve of the Alloy after Double-Ageing Treatment

According to the Hall–Petch formula, σ = σ_0_ + kd^−1/2^, the smaller the grain size of the material is, the greater the number of grain boundaries, and the barrier effect of the grain boundaries on dislocations will increase accordingly with higher strength and hardness. The higher hardness and strength are related to the strengthening effect of the α phase. Lütjering and Sen et al. noted that the hardness and strength are strongly controlled by the grain size of the β phase and α phase as well as the volume fraction of the α phase in titanium alloys [31,32]. The most important factor affecting the mechanical properties of titanium alloys is the α phase. The fine α phase distributed in the β matrix can result in α microstructure conditions similar to that of the α dispersion strengthened system in aged Ti-10V-2Fe-3Al alloys [33].

**Figure 11:**
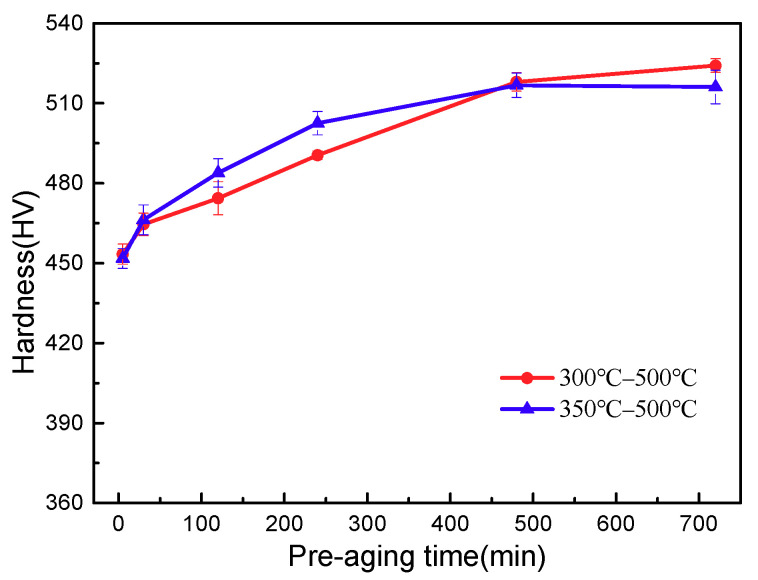
Microhardness of solid solution alloy after double ageing.

The microhardness of the solid solution Ti-3.5Al-5Mo-4V alloy after different double-ageing treatments is displayed in Figure 11. The figure indicates that the hardness value of the alloy increases with the extension of the pre-ageing time, but when the pre-ageing time is short, the pre-ageing temperature hardly affects the hardness of the alloy; this is because when the pre-ageing time is short, the ω phase is less precipitated, and the effect on the later α phase is lessened. When the pre-ageing time is extended from 5 min to 2 h, the hardness of the alloy increases promptly due to the increase in the number of precipitated α phases and the gradual decrease in size, and the higher the pre-ageing temperature is, the faster the hardness of the alloy increases. As the pre-ageing time continues to increase, the hardness of the alloy continues to increase, but the rate of increase is relatively slow, and the hardness of the alloy is the highest at 12 h. The sample treated at 300 °C/12 h + 500 °C/8 h has the most uniform and fine precipitation of the secondary α phase, so its hardness is also the highest, reaching 524 HV (HV0.1).

## 4. Conclusions

In this paper, the microstructural evolution and property change of Ti-3.5 Al-5 Mo-4V alloy were studied by SEM, XRD and TEM. The main conclusions are as follows.

(1) The Ti-3.5Al-5Mo-4V alloy obtains a single coarse grain with an average size of approximately 150 µm after solution treatment at 850 °C/1 h. At 300 °C or 350 °C, for different times of pre-ageing treatment, small ellipsoid-like isothermal ω transitional phases precipitate from the parent β phase with the extension of the pre-ageing time.
(2) The isothermal ω phase exists for shorter and shorter periods as the low-temperature pre-ageing temperature increases. Finally, the isothermal ω phase of the matrix disappears, and the alloy structure is composed of a secondary α phase and β phase. The law of phase precipitation and transformation during low-temperature pre-ageing can be summarised as follows: β → β + ω_iso_ → β + α. When the pre-ageing temperature is constant, the hardness of the alloy increases with the extension of the pre-ageing time and finally becomes stable; when the pre-ageing time is constant, the hardness of the alloy increases with the increase in the pre-ageing temperature.
(3) The secondary α precipitate phase can be effectively refined by double-stage ageing treatment. The α phase under the double-ageing treatment gradually changes from long and thin needles to short needles with the extension of the pre-ageing time and finally changes to an irregular morphology.
(4) In the ascending two-stage ageing process of the alloy, when the pre-ageing time is relatively short, the hardness of the alloy gradually increases with the increase in the pre-ageing temperature. As the pre-ageing time is extended, the hardness of the alloy slowly increases.
(5) The hardness of the alloy is improved by the ascending order two-stage ageing process by refining the secondary α phase. During the pre-ageing process, a large number of secondary α phase nucleation particles (isothermal ω phase or very fine secondary α phase) are formed in the matrix; then, when aged at 500 °C, the secondary α phase is obviously refined due to the existence of nucleation particles. Under the action of external force, the fine secondary α phase can effectively hinder the movement of dislocations, thus improving the mechanical properties of the alloy.

## Figures and Tables

**Table 1:** Chemical composition of Ti-3.5Al-5Mo-4V alloy.

Element	Al	Mo	V	Cr	Sn	Zr	Fe	C	N	O	H	Ti
Mass percent/%	3.62	4.83	3.86	2.09	1.98	2.02	1.01	0.032	0.019	0.007	0.002	Bal.

## Data Availability

Data sharing not applicable.
